# Individual Specialization in a Generalist Apex Predator: The Leopard Seal

**DOI:** 10.1002/ece3.71593

**Published:** 2025-06-23

**Authors:** Emily S. Sperou, Douglas J. Krause, Renato Borras‐Chavez, Patrick Charapata, Daniel P. Costa, Daniel E. Crocker, Kerri J. Smith, Bradley Thompson, Azana Best, Jaelyn Anderson, Michael E. Goebel, Carolina A. Bonin, Sarah S. Kienle

**Affiliations:** ^1^ Department of Biology Baylor University Waco Texas USA; ^2^ Department of Natural Resources Science University of Rhode Island Kingston Rhode Island USA; ^3^ Antarctic Marine Living Resources Program, Ecosystem Science Division National Oceanic and Atmospheric Administration (NOAA) Fisheries La Jolla California USA; ^4^ Center of Applied Ecology and Sustainability (CAPES), Department of Ecology Pontificia Universidad Católica de Chile Santiago Chile; ^5^ Environmental Fisheries Science Division, Northwest Fisheries Science Center National Oceanic and Atmospheric Administration (NOAA) Seattle Washington USA; ^6^ Center for Species Survival Georgia Aquarium Atlanta Georgia USA; ^7^ Department of Ecology and Evolutionary Biology University of California Santa Cruz Santa Cruz California USA; ^8^ Department of Biology Sonoma State University Rohnert Park California USA; ^9^ Department of Biology and Marine Biology University of North Carolina Wilmington Wilmington North Carolina USA; ^10^ Department of Statistical Science Baylor University Waco Texas USA; ^11^ Department of Biological Science Hampton University Hampton Virginia USA

**Keywords:** foraging strategies, individual variation, intraspecific competition, marine mammal, niche variation, specialist

## Abstract

Apex predators are typically considered dietary generalists, which often masks individual variability. However, individual specialization—consistent differences among individuals in resource use or ecological role—is common in apex predators. In some species, only a few specialized individuals can significantly impact prey populations. Leopard seals ( *Hydrurga leptonyx* ) are apex predators important to the structure and function of the Southern Ocean ecosystem. Though broadly described as generalists, little is known about their trophic ecology at the population or individual level. We analyzed δ^13^C and δ^15^N profiles in whiskers (*n* = 46) from 34 leopard seals in the Western Antarctic Peninsula to assess trophic variation. We also evaluated individual consistency across years using repeat samples from 7 seals over 2–10 years. We compared population and individual isotopic niche space and explored drivers of intraspecific variation in leopard seal trophic ecology. We find that leopard seals have a broad trophic niche (range: 6.96%–15.21‰) and are generalists at the population level. However, most individuals are specialists (59% for δ^15^N and δ^13^C), with only a few generalists (13% for δ^15^N, 6% for δ^13^C). Individuals also specialize at different trophic levels. Most variation in trophic ecology is driven by individual specialization, but sex and mass also contribute. We also find that some seals specialize over time, consistently foraging at the same trophic level, while others switch within and between years. This suggests some seals may disproportionately impact prey, especially when specialists consistently target specific species. Long‐term specialization by a few leopard seals likely contributed to the decline of the local Antarctic fur seal population. Our findings show the importance of examining individual specialization in leopard seals across their range to understand their impact on other prey populations. This approach should be applied to other apex predator populations, as a few specialists can significantly impact ecosystems.

## Graphical abstract

Leopard seals are apex predators in the Southern Ocean. The species is broadly described as generalists; however, our study shows that most individual leopard seals are dietary specialists. This specialization has led to niche partitioning among individuals. Individual specialization, drives trophic variation, and we find differences between individuals based on sex and body size. We also find that some leopard seals forage consistently at one trophic level, while others switch trophic levels. In our study location, long‐term specialization by a few large adult female leopard seals has likely contributed to the decline of their prey, Antarctic fur seals. Our study highlights the ecological significance of individual variability in apex predators.

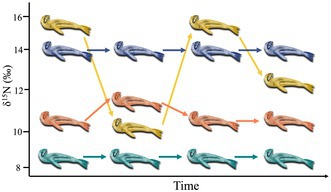

## 1 Introduction

**FIGURE 1:**
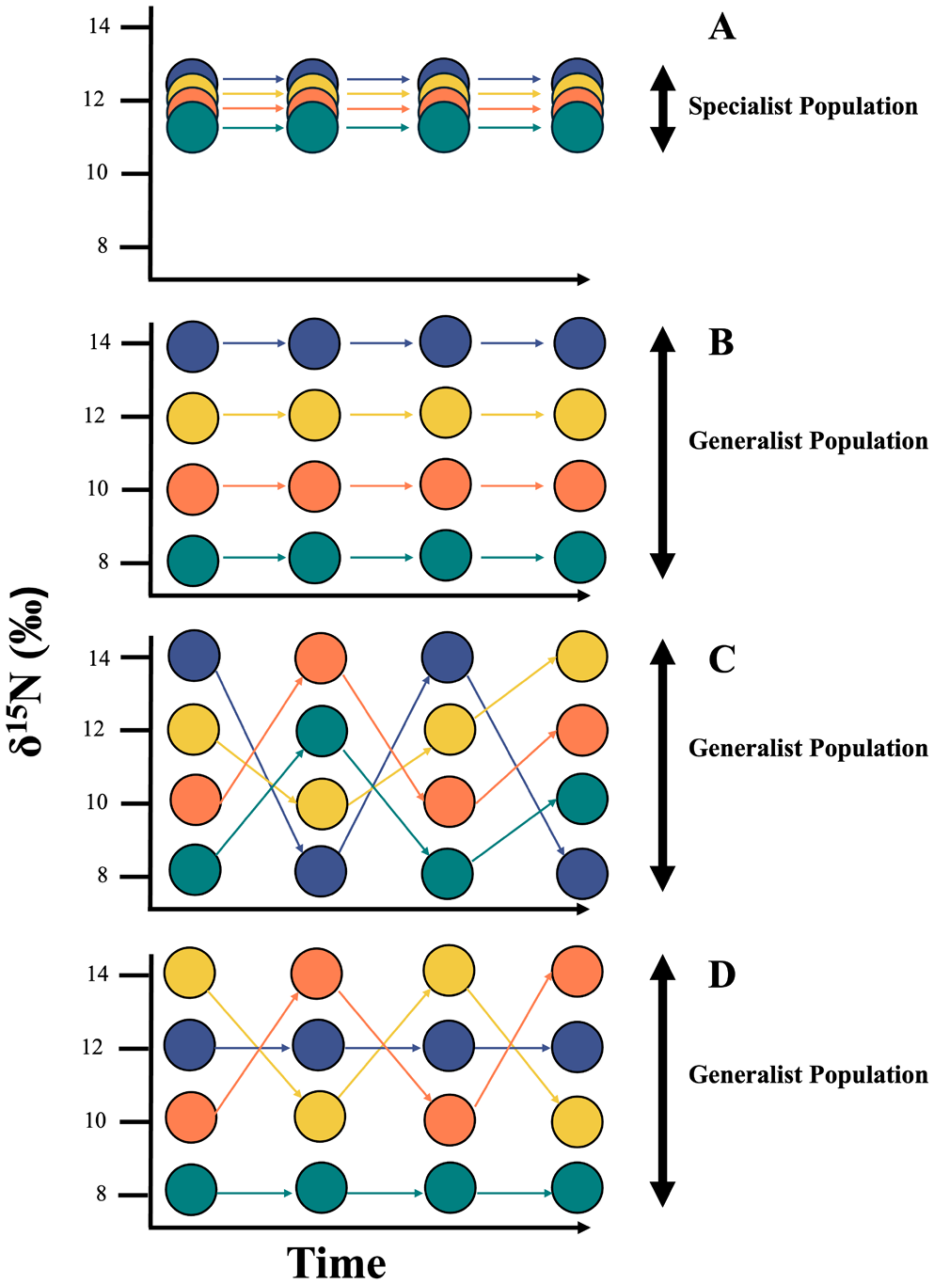
Conceptual model showing four different population‐level patterns of dietary specialization based on isotope signatures of individual diets (δ^15^N) over time adapted from (Vander Zanden et al. 2010). Circles represent individuals and their δ^15^N value for a layer of inert tissue (e.g., whisker, baleen, and claws) reflecting diet. Arrows track changes in individual δ^15^N values through time. (A) A specialist population with a small isotopic niche width composed of four specialist individuals with overlapping δ^15^N values; (B) A generalist population with a large isotopic niche width composed of four different δ^15^N specialist individuals; (C) A generalist population with a large isotopic niche width composed of four generalist individuals; (D) A generalist population with a large isotopic niche width composed of two generalist and two specialist individuals.

**FIGURE 2:**
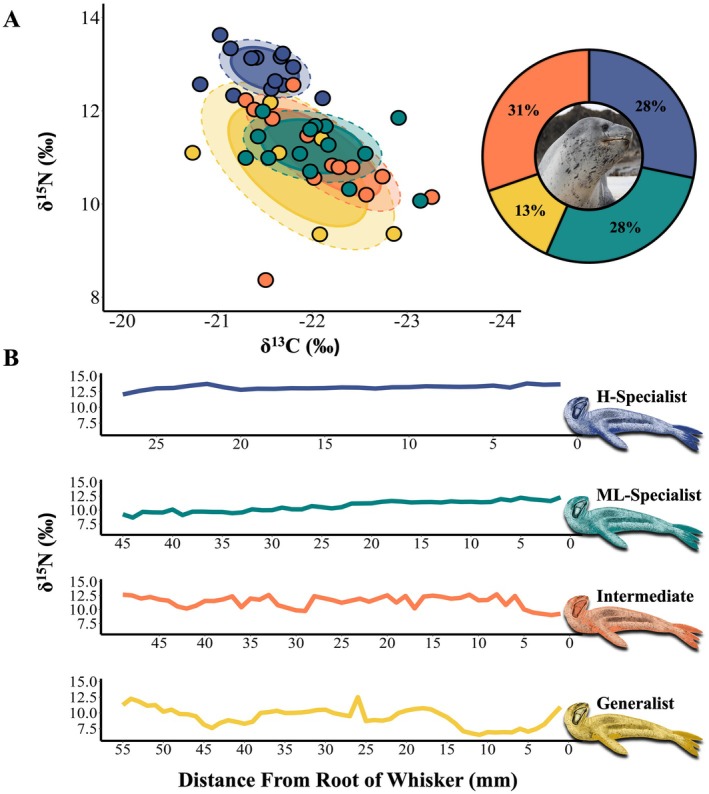
Isotopic analysis of leopard seal whiskers. (A) Population‐level isotopic space (δ^15^N and δ^13^C) for leopard seals color‐coded by δ^15^N specialization category: High trophic‐level specialist (H‐Specialist; blue), medium‐to‐low trophic‐level specialist (ML‐Specialist; green), intermediate (orange), and generalist (yellow). Each point represents the average isotopic value for an individual's whisker(s). Ellipses show standard isotopic ranges for each δ^15^N specialization category: Dashed for 75% and solid for 50% of the data. A pie chart shows the proportion of the population in each δ^15^N specialization category. (B) Representative plots of δ^15^N signatures for a leopard seal whisker of each δ^15^N specialization category (HL‐Specialist: Seal 84; ML‐Specialist: Seal 144; Intermediate: Seal 37; Generalist: Seal 111). Leopard seal art 2024 Roger Hall inkart.net. Leopard seal photo by Renato Borras‐Chavez.

**FIGURE 3:**
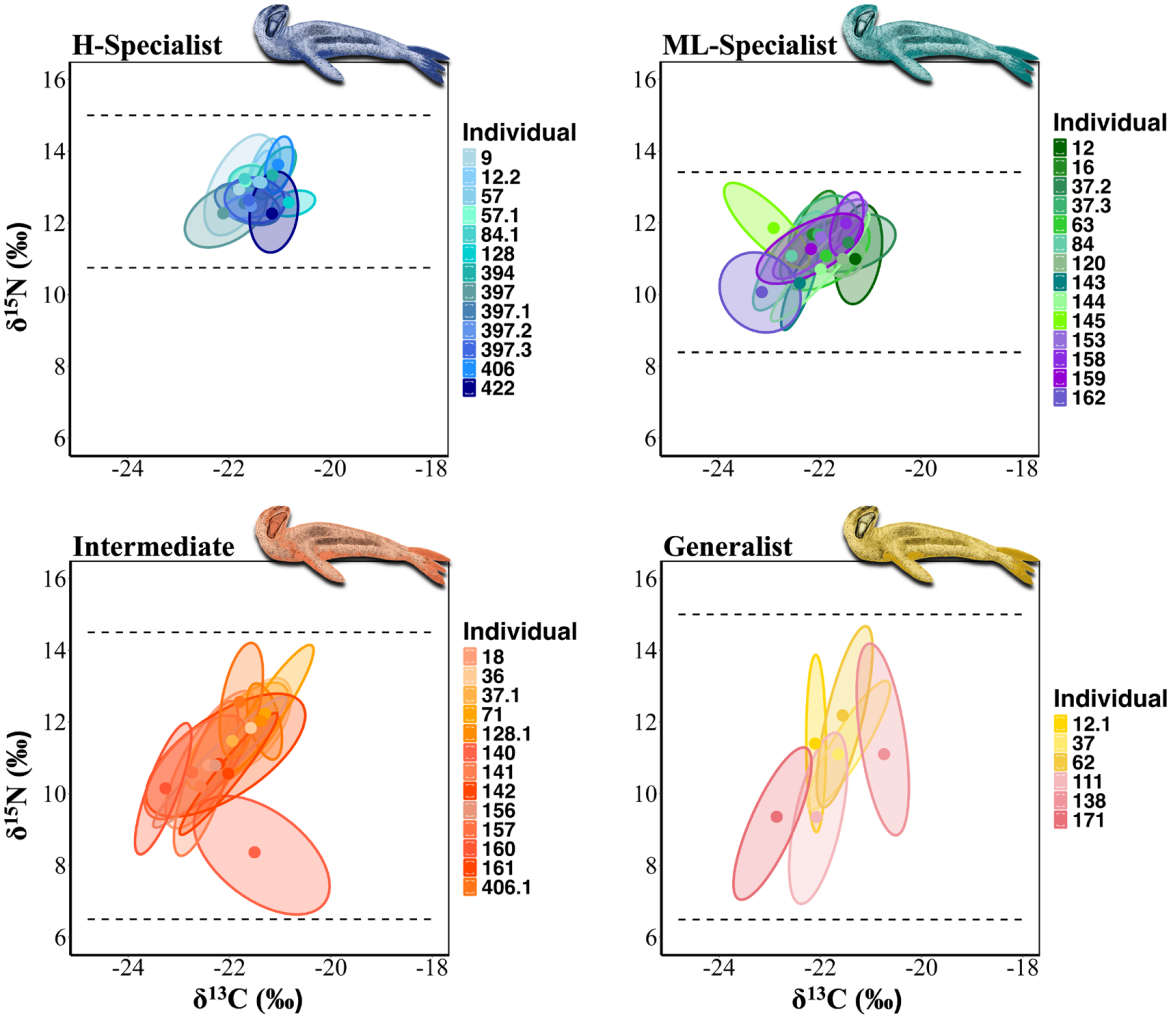
Individual‐level isotopic space (δ^15^N and δ^13^C) for leopard seals color‐coded by δ^15^N specialization category: High trophic‐level specialists (H‐Specialists; blue variations), medium‐to‐low trophic‐level specialists (ML‐Specialists; green and purple variations), intermediates (orange and red variations), and generalists (yellow and pink variations). Ellipses represent the standard isotopic ranges encompassing 75% of the data for each individual's whisker(s), while points indicate the average isotopic value for each individual's whisker(s). The dotted lines represent the δ^15^N range for each category. Leopard seal art 2024 Roger Hall inkart.net.

Classifying species as foraging specialists or generalists can mask individual variability, as individuals within a species, or even within populations, are often not ecologically equivalent (Bolnick et al. 2002; Bearhop et al. 2004; Woo et al. 2008; McPeek and Siepielski 2019). Specialist species have a narrow niche, where individuals consume the same resources with little intraspecific variation (Figure 1A). In contrast, generalist species have a broad niche, consuming a wide range of resources. Generalist populations may consist of: (1) individual specialists who use different resources with little within‐individual but high between‐individual variation (Figure 1B), (2) individual generalists who consume a wide variety of resources with high within‐individual variation (Figure 1C), or (3) a mix of individual specialists and generalists (Figure 1D; Bolnick et al. 2003; Araújo et al. 2011; Hückstädt et al. 2012).

Life history and ecological factors influence intraspecific variation in foraging patterns (Estes et al. 2003; Tinker et al. 2008; Rosenblatt et al. 2015; Jory et al. 2021). For example, foraging patterns vary with sex (Lewis et al. 2006; Elorriaga‐Verplancken et al. 2013; Kernaléguen et al. 2015; Balme et al. 2020), age class (Polis 1984; Thiemann et al. 2007; Balme et al. 2020), location (Staniland et al. 2010; Corman et al. 2016), and/or morphology (Thiemann et al. 2011; Balme et al. 2020; Lewis et al. 2022). Beyond these broad ecological patterns, individuals within a population can also exhibit individual specialization. Van Valen (1965) first emphasized the importance of individual variation in niche theory, arguing that intraspecific differences can shape ecological interactions. Later studies have expanded on this idea by distinguishing between broad intraspecific feeding diversity and true individual specialization, where individuals consistently use distinct subsets of available resources (Bolnick et al. 2003, 2007; Bearhop et al. 2004; Newsome et al. 2009). This differs from intrapopulation feeding diversity, where resource use varies widely at the population level but may not be partitioned among individuals. Individual specialization implies that an individual's niche is significantly narrower than the population's overall niche, with low within‐individual but high between‐individual variation in resource use (Bolnick et al. 2003; Hückstädt et al. 2012; Toscano et al. 2016). This variation reduces competition and enhances population stability during resource fluctuations (Bearhop et al. 2004; Svanbäck and Bolnick 2007; Toscano et al. 2016). Therefore, identifying specialization patterns at both the population and individual levels is crucial for evaluating local and species‐wide patterns of resource use and ecological adaptability.

Individual specialization has been documented across a wide range of taxa, from herbivores to carnivores (Bolnick et al. 2003; Newsome et al. 2009; Riverón et al. 2021; DeSantis et al. 2022). Although widespread, its prevalence and ecological implications can vary depending on an organism's trophic role. Many apex predators and other top predators are considered generalists due to their broad dietary niches at the population level (Matich et al. 2011; Kim et al. 2012; Vejřík et al. 2023). However, apex predator populations are often composed of a mix of both specialists and generalists, as documented in sharks (Matich et al. 2011), leopards (Voigt et al. 2018; Balme et al. 2020), wolves (Darimont et al. 2009), cheetahs (Voigt et al. 2014), and polar bears (Thiemann et al. 2011; Sciullo et al. 2017; King 2024). Individual specialization among apex predators, known for their high energetic demands and foraging efficiency, can significantly impact prey populations, especially when these predators consistently target specific prey species (Williams et al. 2004; Jourdain et al. 2020; Krause et al. 2022). Even a small number of predators can lead to prey population declines (Williams et al. 2004; Pagano et al. 2018; Krause et al. 2022).

Leopard seals ( *Hydrurga leptonyx* ) are important apex predators in the Southern Ocean (Staniland et al. 2018; van der Linde et al. 2021; Krause et al. 2022). They are described as generalists because of their diverse diet that includes endothermic mesopredators (e.g., penguins and other seal species) and ectothermic prey (e.g., Antarctic fish, krill, and cephalopods; Krause et al. 2015, 2020). Furthermore, leopard seals can adapt their feeding strategies based on prey size, using a grip‐and‐tear method for larger prey and employing suction or filtering with their post‐canines for smaller prey (Hocking et al. 2013). Their specialized dentition facilitates this versatility, allowing them to efficiently exploit a wide range of prey across different trophic levels. Leopard seals are also known to exert top–down pressure on prey populations, including Antarctic fur seals (AFS; *Arctocephalus gazella* ; Boveng et al. 1998; Schwarz et al. 2013; Krause et al. 2022) and various penguin species (Ainley et al. 2005; Forcada et al. 2009; Krause et al. 2020).

Cape Shirreff on Livingston Island off the Western Antarctic Peninsula (WAP) is a key feeding ground for leopard seals; it is home to several penguin colonies and the southernmost and regionally largest AFS breeding colony (Hucke‐Gaete et al. 2004). At Cape Shirreff, leopard seals have driven a rapid decline in the AFS population, with an annual average predation rate of ~70% for AFS pups during this study (Vera et al. 2004; Krause et al. 2022). These leopard seals use a variety of specialized prey‐hunting tactics, including stalking, ambushing, and kleptoparasitism (Hiruki et al. 1999; Krause et al. 2015). Despite having access to the same prey resources at Cape Shirreff, single‐point analysis of scat and stable isotope signatures of blood show that these leopard seals show intraspecific variation in diet (Krause et al. 2020; Sperou et al. 2023). Recent work also shows that leopard seal diets vary with sex and body size, with larger females targeting AFS pups and foraging at higher trophic levels than males (Krause et al. 2015, 2020; Sperou et al. 2023). This size advantage may allow large females to specialize and target higher trophic‐level prey compared to conspecifics (Thiemann et al. 2007; Kernaléguen et al. 2015). Therefore, we predict that leopard seals at Cape Shirreff are generalists at the population level but exhibit individual specialization at different trophic levels.

Here, we used stable isotope signatures to investigate population and individual niche width and assess individual specialization in leopard seals. Nitrogen and carbon isotope analyses (δ^15^N and δ^13^C) are commonly used to assess a species' trophic ecology. δ^13^C reflects the source of primary production (e.g., marine vs. terrestrial, pelagic vs. benthic), whereas δ^15^N reflects the trophic level at which individuals forage (Gannes et al. 1998; Tykot 2004). Obtaining individual‐level data on niche width requires the use of accretionary tissues that grow through time (e.g., whiskers, claws, bone, teeth; Bearhop et al. 2004; Elorriaga‐Verplancken et al. 2013; Eisenmann et al. 2016; Lewis et al. 2022; Charapata and Trumble 2023). We used leopard seal whiskers to examine time‐series data from the same individual, representing diet signatures over periods ranging from months to a year. Each segment represents diet intake over time, allowing us to track whether individuals maintain consistent foraging patterns within ~1 year. We then compared isotopic signatures between and within individuals to identify the degree of individual specialization and determine the population and individual niche width (Voigt et al. 2018; Johnson et al. 2022). Using the largest long‐term isotopic dataset on leopard seals to date, we investigated trophic specialization at both the population and individual levels. Specifically, we aimed to address three key questions: (1) What are the patterns of trophic specialization among leopard seals at both population and individual scales? (2) How do phenotypic attributes (e.g., sex, mass) influence foraging strategies? (3) Do individual leopard seals exhibit consistent individual specialization over time?

## 2 Methods

### 2.1 Sample Collection

Leopard seals were sampled between January and May from 2013 to 2023 at the U.S. Antarctic Marine Living Resources (AMLR) Program ecological monitoring site at Cape Shirreff, Livingston Island in the WAP. Leopard seals were sedated (Pussini and Goebel 2015; Krause et al. 2016) to allow for the collection of morphometric data (standard length [cm], girths, and mass [kg]), life history traits (sex, age class), and whiskers. Whiskers were collected from 34 leopard seals (28 females, 6 males). Seven females were opportunistically resampled without a full capture (i.e., morphometric data were not collected) 1–3 times after their first handling, resulting in 12 additional whisker samples. In total, we analyzed 46 whiskers (40 females, 6 males). A subset of these whiskers (*n* = 18 from field seasons 2018 and 2019) were previously analyzed by Charapata et al. (2023).

### 2.2 Stable Isotope Analysis (SIA)

Whiskers were wiped with 1:1 ethanol: methanol solvent, sonicated for 30 min in distilled water, and air dried. Whiskers were measured and sectioned into 0.5–3 mm increments (from root to tip) for a targeted weight of ~0.3 mg (Charapata et al. 2023). Carbon and nitrogen stable isotope analysis was performed at Baylor University using an Elemental Analyzer 4010 Elemental Combustion System paired with a Conflow IV interphase (Thermo Scientific) and Thermo Delta V Advantage continuous flow Isotope Ratio Mass Spectrometer. Whisker nitrogen (δ^15^N) and carbon (δ^13^C) isotope values are expressed in delta notation (δ) in units of per mil (‰). Additionally, isotope values are reported as the ratio of the heavy to light isotope relative to international standards—atmospheric nitrogen and Vienna Peedee Belemnite, respectively—using the following equation:

$$\mathrm{\delta X}=\left[\left(R_{\text{sample}}/R_{\text{standard}}\right)-1\right]\times 1000$$

where X is the 13C or 15N and *R* is the corresponding ratio of 13C/12C or 15N/14N. A two‐point calibration curve for calculating δ^15^N and δ^13^C values of samples was established using USGS‐40 and USGS‐41A international standards. The accuracy and precision of isotopic measurements were calculated based on the long‐term mean and standard deviation (SD) of 244 replicates of an internal lab standard (Acetanilide, reported δ^13^C = −29.53 ± 0.01‰, δ^15^N = 1.18 ± 0.02‰) measured during each analytical run (*n* = 3 replicates/run). The replicate grand averages obtained were very close to (δ^13^C = −29.42 ± 0.08‰) or within the range (δ^15^N = 1.30 ± 0.17‰) of analytical uncertainty of reported values. We measured the atomic C:N ratio for every whisker segment with acceptable atomic ratios ranging from 3.0 to 4.0 (Newsome et al. 2009; Kernaléguen et al. 2012; Charapata et al. 2023). Nearly all whisker segments had acceptable atomic C:N ratios (3.53 ± 0.14, range: 2.9–4.0). Twelve whisker segments were excluded for having ratios outside this range.

### 2.3 Time Stamping

Leopard seals molt and shed their whiskers annually; therefore, whiskers represent growth over a few months and up to one year (Rogers et al. 2016). We timestamped whisker segments based on leopard seal whisker growth characteristics using the Von Bertalanffy growth model (von Bertalanffy 1938; Rogers et al. 2016) following the approach outlined by (Charapata et al. 2023).

### 2.4 Data Analysis

All data were tested for normality and homogeneity of variance before analysis. Results are reported as mean ± SD unless otherwise stated. We performed all analyses using R (R Core Team 2022) with RStudio (Team 2021) and JMP (SAS).

#### 2.4.1 Population Level

Population‐level analyses included a total of 46 leopard seal whiskers. Each whisker was treated separately based on preliminary data showing inter‐annual isotopic variability. We calculated the population‐level mean, SD, and range of δ^15^N and δ^13^C values. We used variance component analysis (VCA) to calculate between‐ and within‐individual population variation. Total variance in stable isotopes (“between individuals” variation) indicates variation among individuals in a population, while variance in stable isotopes along the whisker (“within‐individual” variation) indicates variation of an individual (Bearhop et al. 2004; Newsome et al. 2009; Hückstädt et al. 2012). We applied the Stable Isotope Bayesian Ellipses in the R *SIBER* package (Jackson et al. 2011) to determine population isotopic niche width. We used the standard ellipse area corrected for small sample sizes (SEAc) for individual whisker(s) as the metric for calculating the population isotopic niche area. We also calculated a population‐level SEAc and total area (TA) using the pooled δ^15^N and δ^13^C values from all whisker segments (*n* = 46 whiskers; 2198 segments) to compare our results with a previous study on leopard seals (Botta et al. 2018).

#### 2.4.2 Individual Specialization

We used two approaches to evaluate the isotopic variation at the individual level. First, we calculated individual isotopic niches with SEAc and TA estimates for each individual's whisker(s) using the δ^15^N and δ^13^C values of the whisker segments; this allowed us to visualize and assess each individual's range of trophic levels and foraging locations collectively. Next, we calculated δ^15^N and δ^13^C specialization indices for each whisker to describe the variance in δ^15^N and δ^13^C and calculate the degree of individual specialization (Bolnick et al. 2002; Lewis et al. 2022); this allowed us to separately assess the variation in δ^15^N and δ^13^C. These approaches provide a comprehensive framework to quantify both the breadth of an individual's isotopic niche and the degree of specialization within key isotopic markers, allowing for a more detailed understanding of individual foraging strategies. The degree of specialization was calculated using the equation:

$$\mathrm{SI}=\mathrm{INW}/\left(\mathrm{INW}+\text{BINW}\right)$$

where SI is the specialization index, INW is the individual niche width, and BINW is the between‐individual niche width. Individuals that occupied over 50% of the total isotopic niche width (TNW = INW + BINW; Roughgarden 1972) were classified as generalists (SI > 0.5). Individuals who occupied less than 30% of the total niche width were classified as specialists (SI < 0.3; Bolnick et al. 2003; Hückstädt et al. 2012; Newsome et al. 2015; Lewis et al. 2022). Individuals who occupied between 30% and 50% of the total niche width (0.3 < SI < 0.5) were classified as intermediates (Lewis et al. 2022).

Within the δ^15^N specialist category, some individuals consistently exhibited high δ^15^N values, while others consistently had medium‐to‐low values. Therefore, we performed agglomerative hierarchical clustering to determine whether there were subgroups within our δ^15^N specialist isotope data using the “agnes” function in the R package *cluster* (Kaufman and Rousseeuw 2009). To determine the optimal number of clusters, we used the Dunn index, which differentiates between sets of clusters that are compact and well separated (Figure S1A). We found two distinct clusters (Figure S1B): high trophic‐level specialists (H‐Specialist) and medium‐to‐low trophic‐level specialists (ML‐Specialist).

#### 2.4.3 Trophic Variation and Overlap

We examined variation and niche overlap in isotopic signatures as a function of sex, body mass, and degree of individual specialization. We focused these analyses solely on δ^15^N because (1) we were interested in trophic‐level variability, and (2) leopard seals from Cape Shirreff tend to remain in the near‐shore habitat and are primarily coastal foragers (Krause et al. 2015; Kienle, et al. 2022). To investigate variation in δ^15^N, we ran a linear mixed‐effects model (LMM; Pinheiro and Bates 2000) using the “lmer” function from the *lme4* package (Bates et al. 2014). This model treated the average δ^15^N values as response variables with sex, mass, the interaction of sex and mass, and δ^15^N specialization category (H‐Specialist; ML‐Specialist; Intermediate; Generalist) as fixed effects and individual as a random effect to account for repeated sampling of some individuals. We initially tested year as a factor but, as it had little effect on the model, we opted for the simpler model and analyzed temporal variation separately using a GAM, which is better suited for detecting trends over time, particularly when dealing with uneven sample sizes across years. All 46 whiskers were included in the analysis to ensure comprehensive representation across individuals. We assessed all model assumptions prior to analysis to ensure statistical validity. Model selection was performed using the R package *MuMIn* (Barton and Barton 2015) based on the smallest Akaike information criterion corrected for sample size (AICc). The model with the lowest AICc had the highest support, and models with ΔAICc < 2 were considered to have substantial support (Anderson and Burnham 2002; Franklin et al. 2002). Goodness‐of‐fit for each model was estimated using marginal (*R* ^2^ LMM(m)) and conditional (*R* ^2^ LMM(c)) coefficients of determination, indicating variance explained by fixed effects alone and by both fixed and random effects, respectively (Nakagawa and Schielzeth 2013). We examined the contribution of each fixed effect of our top models by looking at the estimated coefficients and *p*‐values and then used ANOVAs on each of our top models. Pairwise comparisons were performed using the “emmeans” function from the *emmeans* package with Tukey adjustment for multiple testing δ^15^N and δ^13^C (Lenth et al. 2022). We also used Spearmen's correlation to assess the relationship between our continuous variables (average δ^15^N, mass) and the relationship between mass and individual niche width (SEAc). We assessed niche differences and overlap between δ^15^N specialization categories and sexes using the proportion of paired SEAc shared; this was calculated with the “maxLikOverlap” function from the *SIBER* package (Jackson et al. 2011).

#### 2.4.4 Between‐Year Variability

To evaluate between‐year variability among repeat individuals (*n* = 7), we used *SIBER* to visualize data and quantify percent overlap between isotopic niches, assessing the similarity/dissimilarity in isotopic composition between years for each individual. We also used a quadratic discriminant analysis (QDA) to simultaneously evaluate δ^13^C and δ^15^N (Koehler et al. 2019; Smith et al. 2021). QDA is appropriate for analyzing data that are unequally sampled across years and have unequal variance; this allowed us to effectively assign isotope signatures to specific years for each seal. We considered QDA to be unsuccessful in assigning individual isotope data to their respective years if the results were ≤ 70% (Koehler et al. 2019; Smith et al. 2021), suggesting that the data was too similar to accurately assign it to specific years.

#### 2.4.5 Temporal Changes

To investigate yearly and monthly trends in δ^15^N data, we used generalized additive models (GAMs) with isotopic signatures of whisker segments as the response variable. Year and month from timestamped whiskers were used as temporal predictors, with the individual as a random effect, using the formula: δ^15^N ~ s(Months, *k* = 10, bs = “cc”) + s(Year, *k* = 10, bs = “tp”) + s(Individual.ID, bs = “re”). The “s” represents the smooth functions and “k” represents the number of basis functions used in the smoothing function. For a month we used a cyclic spline (bs = “cc”) and for a year a thin plate regression spline (bs = “tr”; Wood 2003). GAMs and corresponding model estimates were conducted using the *mgcv* and *modelbased* R packages (Wood 2017; Makowski et al. 2020). We evaluated isotopic linear temporal fluctuations in significant variables by using a grid approximation, accompanied by a CI of 95% (Makowski et al. 2020). Because our dataset is overrepresented in some years/months and underrepresented in others, we created a customized prediction grid based on GAM models fitted to the observed data. The conditional expectations generated by the simulated homogeneous dataset (i.e., simulated data for all years and months for all Individual.ID) allowed parameter estimation using the fitted model. The predicted values were used to calculate the first derivative of the response variable and estimate the linear slope of the isotopic signatures to identify the temporal windows where significant linear increases or decreases of isotopic values occurred in time.

## 3 Results

We sectioned 46 whiskers and analyzed 2198 segments for δ^13^C and δ^15^N (Table 1). The average number of segments per whisker was 47.8 ± 18.06 (range: 6–89 segments). Timestamped whiskers represented 99.5 ± 52 days (range: 19–286 days), which is consistent with previous estimates (max ~1 year) of leopard seal whisker growth (Rogers et al. 2016).

**TABLE 1:** Summary of isotopic values for leopard seals (34 individuals, 46 whiskers) categorized based on the δ^15^N category: High trophic‐level specialist (H‐Specialist), medium‐to‐low trophic‐level specialist (ML‐Specialist), intermediate, and generalist.

ID	Sex	Year	*n*	*δ* ^15^N	SD	Range	*δ* ^13^C	SD	Range	SEAc ‰^2^
**H‐Specialist**										
9	F	2014	51	12.94	0.89	10.88–14.21	−21.79	0.46	−22.83 to −20.94	1.16
12*	F	2018	61	13.14	0.63	10.88–13.87	−21.41	0.19	−21.98 to −20.92	0.29
57*	F	2018	45	13.13	0.83	10.92–14.05	−21.36	0.27	−22.13 to −20.82	0.57
	F	2023	6	13.16	0.30	12.69–13.63	−21.67	0.23	−21.87 to −21.26	0.23
84*	F	2017	26	13.23	0.28	12.64–13.82	−21.68	0.29	−22.49 to −21.37	0.26
128*	F	2014	44	12.58	0.28	12.02–13.66	−20.81	0.44	−22.78 to −20.37	0.38
394	F	2013	22	13.34	0.49	12.12–13.92	−21.14	0.30	−21.94 to −20.68	0.43
397*	F	2014	77	12.27	0.65	11.01–13.97	−22.11	0.54	−23.30 to −21.30	1.00
	F	2018	46	12.55	0.41	11.66–13.49	−21.69	0.46	−23.06 to −20.97	0.57
	F	2019	62	12.47	0.31	11.02–12.97	−21.56	0.59	−23.82 to −20.87	0.48
	F	2023	49	12.63	0.45	11.64–13.62	−21.60	0.50	−23.03 to −20.89	0.73
406*	F	2013	71	13.63	0.57	12.35–15.21	−21.02	0.40	−22.30 to −18.71	0.63
422	F	2013	59	12.26	0.76	10.26–13.43	−21.16	0.40	−22.85 to −20.47	0.93
**ML‐Specialist**										
12*	F	2013	47	10.98	0.93	8.60–12.26	−21.29	0.38	−22.73 to −20.77	1.15
16	F	2014	36	11.67	0.85	10.22–13.41	−22.14	0.32	−22.85 to −21.51	0.75
37*	F	2019	47	11.45	0.56	9.78–12.47	−21.43	0.59	−22.96 to −20.56	1.02
	F	2023	35	11.67	0.73	10.17–13.36	−22.02	0.50	−23.26 to −21.35	1.18
63	F	2014	52	11.08	0.73	8.51–12.56	−21.87	0.55	−22.80 to −20.81	1.21
84*	F	2014	23	11.07	0.89	9.92–12.61	−22.55	0.47	−23.09 to −21.59	0.82
120	M	2017	18	10.98	0.41	10.06–11.78	−21.54	0.35	−22.60 to −21.03	0.48
143	F	2018	29	10.31	0.87	8.93–12.53	−22.39	0.27	−23.06 to −21.90	0.69
144	M	2018	44	10.70	0.97	8.65–12.29	−21.97	0.60	−23.11 to −20.92	0.82
145	F	2018	64	11.85	0.79	10.17–13.11	−22.91	0.60	−23.69 to −21.69	1.20
153	F	2019	48	11.60	0.75	10.48–13.38	−21.98	0.58	−22.87 to −20.82	0.93
158	F	2019	44	11.98	0.59	10.51–12.90	−21.48	0.24	−22.19 to −20.98	0.43
159	F	2019	43	11.27	0.64	9.70–12.69	−22.16	0.65	−23.20 to −21.02	1.19
162	F	2019	74	10.07	0.77	8.39–11.81	−23.14	0.56	−24.76 to −21.89	1.41
**Intermediate**										
18	F	2014	46	10.79	1.25	7.34–12.54	−22.42	0.59	−23.75 to −21.46	1.61
36	F	2013	89	11.83	1.04	8.29–13.35	−21.58	0.59	−23.74 to −20.67	1.38
37*	F	2014	44	11.47	1.03	9.01–12.73	−21.95	0.72	−23.99 to −20.97	1.72
71	F	2013	27	12.23	1.23	9.42–13.95	−21.30	0.57	−22.43 to −20.52	1.03
128*	F	2023	47	12.03	1.12	7.82–15.17	−21.38	0.33	−22.68 to −20.93	1.21
140	M	2018	56	8.36	1.07	6.95–11.68	−21.51	0.87	−23.46 to −20.36	2.73
141	M	2018	60	10.20	1.38	7.74–12.14	−22.57	0.50	−24.25 to −21.96	1.56
142	F	2018	44	10.83	1.23	7.61–12.31	−22.21	1.02	−24.43 to −20.70	3.28
156	F	2019	77	10.79	1.19	8.28–13.23	−22.28	0.84	−23.99 to −20.87	1.06
157	M	2019	71	10.59	1.02	8.02–11.89	−22.74	0.64	−23.95 to −21.30	1.64
160	F	2019	73	10.15	1.16	7.93–11.87	−23.26	0.43	−23.32 to −20.83	1.01
161	F	2019	69	10.56	1.22	7.96–14.13	−22.02	0.66	−23.32 to −20.83	0.80
406*	F	2014	56	12.56	1.06	10.25–14.28	−21.79	0.28	−22.55 to −21.22	0.87
**Generalist**										
12*	F	2017	22	11.39	1.58	8.26–14.04	−22.10	0.13	−22.44 to −21.88	0.71
37*	F	2013	36	11.09	1.41	7.54–12.87	−21.65	0.66	−23.07 to −21.04	1.85
62	F	2023	25	12.18	1.59	9.40–14.38	−21.56	0.38	−22.41 to −20.93	1.58
111	M	2017	53	9.35	1.53	6.49–12.51	−22.07	0.37	−23.05 to −21.52	1.60
138	F	2018	30	11.10	1.95	6.96–13.80	−20.74	0.48	−22.50 to −19.83	2.90
171	F	2020	51	9.35	1.48	7.16–12.82	−22.86	0.53	−23.77 to −21.70	1.60

*Note:* “*n*” is the number of whisker segments analyzed. “SEAc” is the standard ellipse area corrected for small sample sizes for each individual's whisker(s). Seals with an asterisk (*) were sampled in multiple years.

### 3.1 Population Level

Leopard seals were classified as a generalist population, with a large population‐level SEAc of 3.35‰^2^ and TA of 39.82‰^2^. Mean δ^13^C whisker isotopic signature was −21.93‰ ± 0.8‰ (range: −24.76% to −18.71‰), and mean δ^15^N was 11.46‰ ± 1.53‰ (range: 6.49‰–15.21‰). Between‐individual variability was higher than within‐individual variability in both δ^13^C (53% vs. 47%, respectively) and δ^15^N (58% vs. 42%, respectively).

### 3.2 Individual Specialization

Our two approaches to assess specialization (i.e., individual isotopic widths and specialization index) showed that most individuals were specialists (Table 1; Figures 2 and 3). The mean individual SEAc was 1.12‰^2^ ± 0.65‰^2^ (range: 0.23‰^2^–3.28‰^2^). The mean TA was 4.16‰^2^ ± 2.13‰^2^ (range: 0.26‰^2^–9.93‰^2^). The results of our specialization index and individual isotopic width analysis were consistent: individuals with SEAc values > 2 were identified as δ^15^N or δ^13^C generalists, while those with SEAc values < 1 were classified as δ^15^N or δ^13^C specialists.

Based on the δ^15^N specialization index, 59% of leopard seals (*n* = 27) were specialists, 28% (*n* = 13) were intermediates, and 13% (*n* = 6) were generalists (Figure 2A), with specialists showing little δ^15^N isotopic variation and generalists exhibiting high δ^15^N isotopic variation (Figure 2B). Within the δ^15^N specialists, there were two separate clusters: H‐Specialists and ML‐Specialists. Thirteen seals were H‐Specialists (mean δ^15^N = 12.90 ± 0.42‰; range: 10.26%–15.21‰). Fourteen seals were ML‐Specialists (mean δ^15^N = 11.19 ± 0.54‰; range: 8.39%–13.41‰). The δ^15^N intermediates had a mean δ^15^N of 10.95‰ ± 1.10‰ (range: 6.95%–14.28‰), and δ^15^N generalists had a mean δ^15^N of 10.74 ± 1.15‰ (range: 6.49%–14.04‰). A similar pattern was observed for δ^13^C: 54% (*n* = 25) were specialists, 37% (*n* = 17) were intermediates, and 6% (*n* = 3) were generalists.

The isotopic niche spaces for all individuals, classified by their respective δ^15^N specialization categories, are shown in Figure 3.

### 3.3 Trophic Variation and Overlap

Our top models showed that δ^15^N varied by sex, mass, δ^15^N specialization category, and individual (Tables S1 and S2). Our top model had an *R* ^2^ (c) of 0.58 and an *R* ^2^ (m) of 0.92, indicating that individuals accounted for a significant portion of the model variation. The results of our top models and overlap analysis are detailed below:

- *δ* ^*15*^ *N Specialization*. Mean δ^15^N differed between specialization categories (*F*_3,17.37_ = 23.01, *p* < 0.001). H‐Specialists had a higher δ^15^N (12.90‰ ± 0.4‰; *p* < 0.001) compared to other groups. ML‐Specialists had the next highest δ^15^N (11.19‰ ± 0.56‰), followed by intermediates (10.95‰ ± 1.0‰) and generalists (10.74‰ ± 1.15‰). Isotopic niche areas, SEAc, and TA values were the smallest for H‐Specialists (SEAc = 0.49; TA = 1.05‰^2^) and largest for generalists (SEAc = 2.55‰^2^; TA = 2.80‰^2^; Figures 2A and 3). There was no niche overlap between H‐Specialists and other groups. However, ML‐Specialists, intermediates, and generalists showed 27%–95% overlap (Figure 2A).
- *Sex Differences*. Mean δ^15^N varied with sex (*F*_1,30.66_ = 11.84; *p* = 0.001). Females had higher δ^15^N values (11.71‰ ± 1.39‰) than males (9.92‰ ± 1.46‰; *F*_1,2197_ = 423.3, *p* < 0.001). The TA was larger in females (6.24‰^2^) than in males (1.69‰^2^) and was driven primarily by δ^15^N (Figure S2A). Females had a slightly smaller SEAc (1.44‰^2^) compared to males (1.91‰^2^). Females showed greater inter‐individual variation in an isotopic niche (individual δ^15^N means from 9.4% to 13.63‰; δ^13^C means from −23.26% to −20.74‰) compared to males (individual δ^15^N means from 8.37% to 10.99‰, δ^13^C means from −22.74% to −21.51‰). Isotopic niche overlap between males and females was small (range: 7.5%–10.0%; Figure S2A).
- *Body Mass*. Mean δ^15^N varied with mass (*F*_1,5.04_ = 8.84; *p* = 0.03). Larger seals had higher δ^15^N values than smaller seals (*R* ^2^ = 0.28, *p* < 0.05; Figure S2B). We found a positive association between δ^15^N and mass for females (Spearman's ρ = 0.40, S = 4259.2, *p* = 0.01) and males (Spearman's ρ = 0.829, S = 6, *p* = 0.05). Lastly, we found a negative relationship between mass and SEAc, with larger individuals having smaller SEAc values than smaller individuals (*F*_1,39_ = 10.3, *p* = 0.002; Figure S2C).

### 3.4 Between‐Year Variability

**FIGURE 4:**
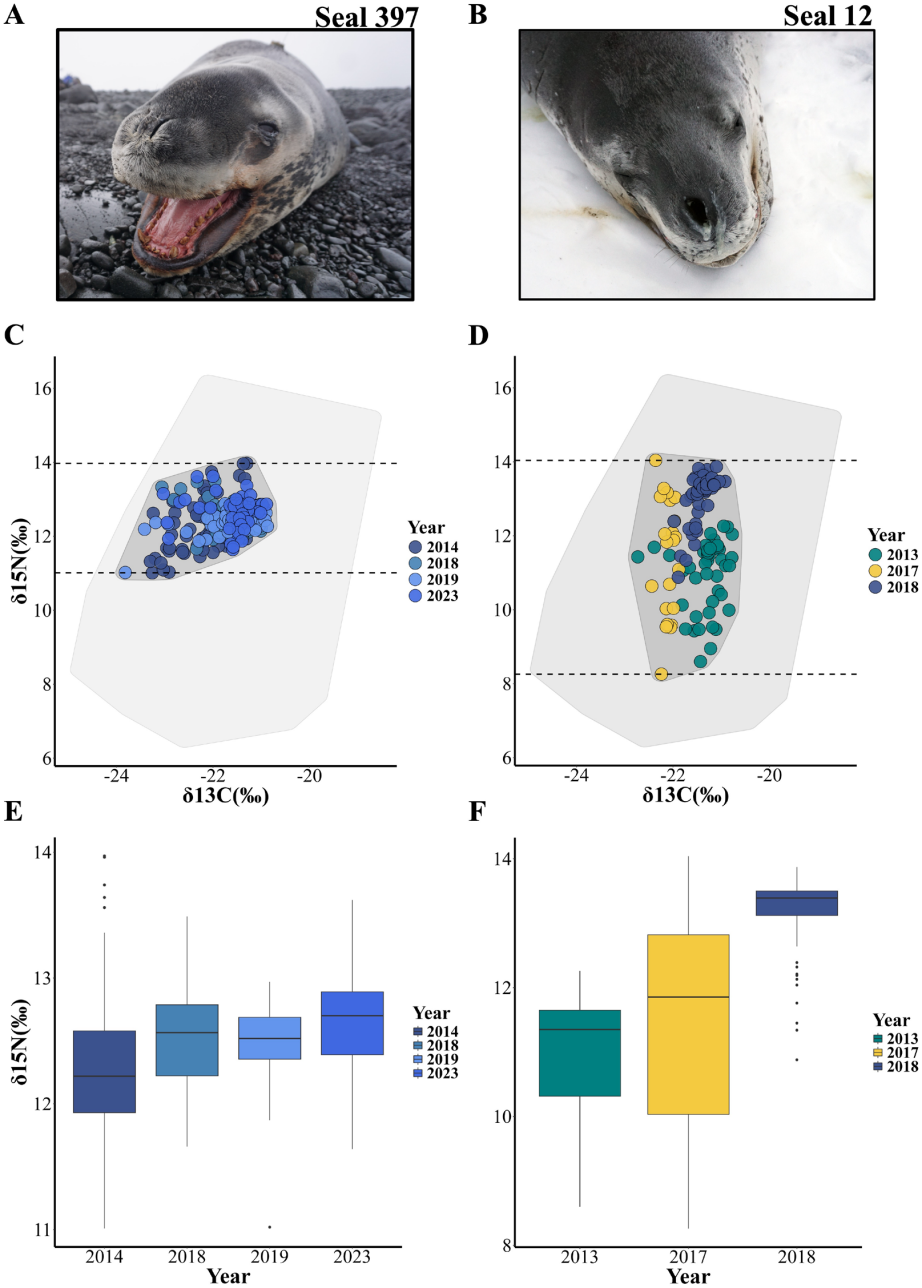
Comparison of isotopic consistency between a long‐term δ^15^N H‐S for specialist (A; Seal 397) and a variable specialist (B; Seal 12) across different years. Bivariate plots of isotopic space (δ^15^N and δ^13^C) for Seal 397 (C), showing consistency in δ^15^N values across the years, and for Seal 12 (D), showing variation in δ^15^N values across the years. Each point represents an individual whisker segment. The dark gray polygon shows the individual's total isotopic space. The light gray polygon shows the population's total isotopic space. The colors represent the δ^15^N specialization category assigned each year: High δ^15^N specialists (H‐Specialists) in blues, medium‐to‐low specialists (ML‐Specialists) in green, and generalists in yellow. Box and whisker plots of δ^15^N values for Seal 397 (E) and Seal 12 (F) show examples of consistency (E) in δ^15^N isotope values across different years compared to variability associated with prey switching (F). In both plots, horizontal bars represent the mean concentrations for each individual and the ends represent the range. Leopard seal photos by Dan Costa.

Between‐year variability exhibited two distinct patterns (Figure 4; Figure S3). Some seals had consistent foraging patterns. These seals had high overlap in isotopic niche space between years, making assigning isotopic values by year challenging. For example, seal 397 had high isotopic niche overlap over a 10‐year period (54%, [23%–91%]; Figure 4) and QDA was unsuccessful (< 70%) at assigning isotopic values to different years (45%, [28%–60%], Tables S3 and S4). Conversely, other seals showed considerable variability in foraging patterns between years. These seals showed little to no overlap in isotopic niche space; this allowed us to successfully assign their isotopic values to different years. For example, seal 12 showed no overlap between years, and QDA was successful at assigning isotopic values to the correct year (96%, [92%–100%], Figure 4, Tables S3 and S4).

### 3.5 Temporal Trends

**FIGURE 5:**
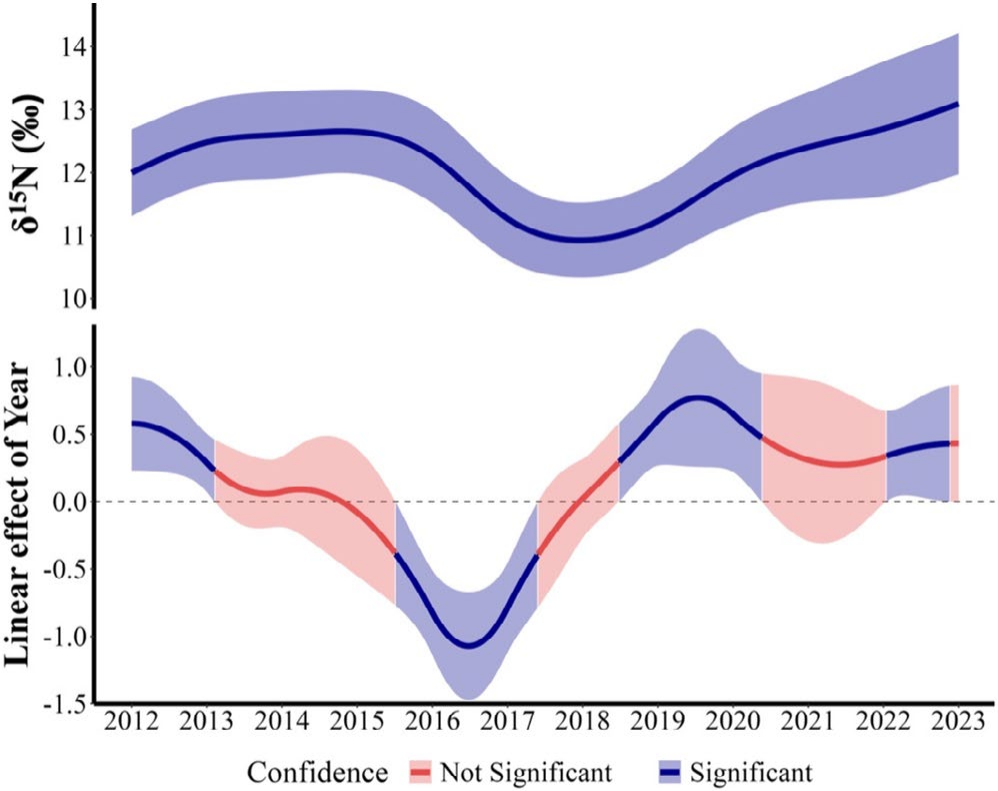
Yearly trends in δ^15^N values for leopard seals (2012–2023). Top panel shows the yearly δ^15^N values with the fitted trend (blue line) and the 95% confidence interval (shaded area). Bottom panel shows the slope of the seasonal rate of change in the predicted δ^15^N values based on the first derivative of the fitted GAM shown in the upper panel. The bottom panel displays temporal windows where significant linear increases or decreases of isotopic values occurred in time, with the red line representing the fitted trend. Blue and red shaded areas indicate non‐significant and significant effects, respectively, at the 95% confidence level.

Temporal variation in δ^15^N was non‐linear. There was a significant non‐linear δ^15^N variation explained by year (*F*_6.95,7.81_ = 7.03, *p* < 0.001) but not by months (Table S4). Year explained some model variance (0.42 [95% CI: 0.31–1.37]). However, most of the model variance (1.30 [95% CI: 0.91–1.4]) was from the random effect (*F*_42.34,45_ = 51.43, *p* < 0.001), representing intraspecific variability. Analytic estimation of the first derivative calculated from the grid approximation showed temporal windows of significant linear decrease and increase in δ^15^N throughout the years (Figure 5). From these significant temporal windows, we highlight the decline of δ^15^N from early 2015 to mid‐2017 calculated by our estimation (linear slope β = −0.8, 95% CI [−1.15–0.44]).

## 4 Discussion

Individual specialization in apex predators can shape ecosystems by impacting prey populations and resource use. Leopard seals at Cape Shirreff are a generalist population predominantly composed of individual specialists. They show clear niche partitioning between specialization categories (i.e., specialists, intermediates, and generalists) and consistent overlap among individuals within those categories. Individual differences are the primary driver of intraspecific variation in leopard seal trophic ecology, but life history traits (i.e., sex and mass) also affect trophic variability. Additionally, some leopard seals consistently specialize across years, while others switch between foraging strategies across years. This is the first study to evaluate individual foraging patterns in leopard seals over extended periods (days to years) using the largest time‐series dataset available for this apex predator. As such, our results advance our understanding of apex predators' trophic ecology at both the population and individual levels, contributing to broader insights into their ecological role. Our findings emphasize the importance of individual diet assessments for apex predators, as only a few individuals disproportionately impact specific prey.

### 4.1 Population and Individual Level

Leopard seals are described as generalist predators due to their diverse diets (Hall‐Aspland and Rogers 2004; Botta et al. 2018; Krause et al. 2020). Our population‐level results at Cape Shirreff show leopard seals occupy a broad isotopic niche, primarily driven by their wide range in δ^15^N values. This finding complements dietary studies showing that leopard seals at Cape Shirreff consume diverse prey, including AFS, penguins, fish, cephalopods, and krill (Krause et al. 2020). Their unique dental morphology and ability to employ multiple prey capture strategies provide them with the behavioral flexibility necessary to exploit a wide range of trophic resources efficiently (Hocking et al. 2013; Kienle and Berta 2016).

However, like many other apex predators and other species of pinnipeds (Hückstädt et al. 2012; de Lima et al. 2019; Riverón et al. 2021), leopard seals exhibit a high degree of individual specialization. At Cape Shirreff, most (87%) of leopard seals are δ^15^N specialists or intermediates, while only a few are true δ^15^N generalists (13%). Consequently, individual leopard seals have relatively narrow niche widths, despite the population having a large overall niche width. Furthermore, among the δ^15^N specialists, leopard seals show resource partitioning; some specialize in high trophic‐level prey, while others specialize in medium‐to‐low trophic‐level prey. The high trophic‐level specialists are likely foraging on AFS pups and/or large notothen fish, while the medium‐to‐low specialists are likely foraging on penguins, cephalopods, fish, or krill (Botta et al. 2018; Krause et al. 2020). This partitioning likely reduces direct competition among individuals and enhances individual foraging efficiency. As we hypothesized, leopard seals at Cape Shirreff collectively have a large population isotopic niche, but exhibit a high degree of individual specialization, leading to relatively narrow and overlapping niches with distinct partitioning between trophic groups.

### 4.2 Drivers of Individual Specialization

Resource diversity and competition are known to influence the development of individual‐based specialized foraging strategies in many predators (Estes et al. 2003; Layman et al. 2007; Darimont et al. 2009; Weise et al. 2010; Manlick et al. 2021). For example, some Asian predators (e.g., dholes, leopards, tigers) exhibit specialization in prey‐rich areas but prey shift in areas with limited resources (Steinmetz et al. 2021). Similarly, we find that individual specialization explains most of the intraspecific variation in leopard seal trophic ecology. We suggest that specialization by leopard seals at Cape Shirreff is driven by the combination of a prey‐rich environment and intraspecific competition for high trophic‐level prey.

During the study period (2011–2020), Cape Shirreff was home to ~20 seasonal‐resident leopard seals (range: 11–41), primarily adult females, with peak numbers in the austral summer (Krause et al. 2022). Despite the variety of prey available around Cape Shirreff, leopard seals appear to preferentially target energy‐rich, endothermic prey, such as AFS pups and fledging penguins (Spitz et al. 2014; Krause et al. 2015, 2020; Raga et al. 2015; Hinke et al. 2019; Lu et al. 2021). These prey are easily available during the mesopredators' breeding season, driving resource competition among leopard seals and the adoption of individual foraging strategies. To capture prey, leopard seals employ a variety of specialized hunting tactics (Hiruki et al. 1999; Krause et al. 2015) and large adult females often outcompete smaller individuals and even steal prey from conspecifics (Krause et al. 2015, 2020; Sperou et al. 2023). For instance, seal 397, a large adult female, has the highest observed capture rate of AFS pups using an intertidal ambush technique, while other leopard seals simultaneously employed different tactics to target other prey (Krause et al. 2015). Intraspecific competition for these energy‐rich resources likely facilitates individual specialization and widens the leopard seals' trophic niche.

In many apex predators, larger individuals (often males) have more varied diets and consume higher trophic‐level prey than smaller individuals (Thiemann et al. 2007, 2011; Kernaléguen et al. 2015; Voigt et al. 2018; de Lima et al. 2019; Balme et al. 2020). Similar patterns have been described in leopard seals, where the larger sex (females) outcompetes smaller individuals of both sexes, especially in competition for large endothermic prey (Krause et al. 2015, 2020; Sperou et al. 2023). At Cape Shirreff, all larger seals are females, resulting in sex‐based differences in trophic level and minimal overlap between sexes. Females occupy a broader isotopic space, forage on higher trophic‐level prey, and are more often specialists compared to males. Moreover, all high trophic‐level specialists in this study were female. Therefore, larger body sizes seem to provide access to a wider array of higher‐quality prey (Svanbäck and Bolnick 2007; Araújo et al. 2011; Balme et al. 2020; Kienle, Friedlaender, et al. 2022; Lewis et al. 2022; this study).

Further south in the WAP, Botta et al. (2018) examined leopard seals' isotopic niche at Danco Coast. Similar to this study, the authors showed that leopard seals had a broad population‐level isotopic niche width and that some individual seals consistently had high or low δ^15^N values along their whiskers (Botta et al. 2018). However, Danco Coast leopard seals occupied lower trophic levels (mean δ^15^N = 8.9‰, range: 6.6%–12.0‰) compared to Cape Shirreff seals (11.46‰, range: 6.4%–15.2‰). This is likely due to prey differences. At Danco Coast, leopard seals are primarily consuming lower trophic‐level prey (e.g., krill, cephalopods, and small fish; Casaux et al. 2009; Botta et al. 2018). In comparison, Cape Shirreff offers greater prey diversity and an abundance of higher trophic‐level prey (Krause et al. 2020). Additionally, our study included mostly females, while Botta et al. (2018) mostly included males. Therefore, in addition to prey availability, the trophic‐level differences between leopard seals at Cape Shirreff and Danco Coast may also be due to sex (and size) differences between the two aggregations.

### 4.3 Long‐Term Specialization

Some leopard seals are extremely consistent in their foraging patterns, while others are highly flexible. For example, adult female seal 397 remained a high trophic‐level specialist over a 10‐year period (this study), and primarily targeted AFS pups (Krause et al. 2015). Conversely, adult female seal 12 switched from a high trophic‐level specialist to a medium‐to‐low trophic‐level specialist to a generalist over a 5‐year period. Similar patterns have been documented in other marine predators (McHuron et al. 2018). For instance, southern rockhopper penguins ( *Eudyptes chrysocome* ) change diets seasonally, acting as specialists during pre‐breeding and generalists during pre‐molt (Dehnhard et al. 2016). In other species, individual specialization persists over longer periods. For example, southern elephant seals ( *Mirounga leonina* ), show consistent foraging patterns within and between years (Hückstädt et al. 2012). Alternatively, some species show a lot of variability. In California sea lions ( *Zalophus californianus* ), individuals remain consistent in their diet while others switch diets annually due to ecological shifts (McHuron et al. 2018). Collectively, these findings suggest that some marine predators, including leopard seals, can exist along a continuum from consistent specialists to highly flexible generalists.

The variation in foraging specialization among leopard seals raises the question of whether they are true specialists or flexible generalists. Specialists typically exploit a narrow dietary niche more restricted than the population's (Bolnick et al. 2003). While some leopard seals fit this definition—such as those consistently preying high trophic‐level prey (such as AFS)—others shift their foraging strategies over time, suggesting foraging flexibility.

This variability may indicate that leopard seals are context‐dependent foragers, adjusting their diet based on prey availability, reproductive status, or environmental conditions. Rather than being strict specialists, some individuals may switch between specialist and generalist strategies as needed. Similar patterns occur in other apex predators, such as orcas ( *Orcinus orca* ), where transient populations specialize in marine mammals while others exhibit greater dietary flexibility (Williams et al. 2004; Jourdain et al. 2020).

Foraging strategy variability may buffer populations against rapid environmental changes. In northern elephant seals ( *M. angustirostris* ), females with high site fidelity to particular foraging locations had higher foraging success in average climate conditions; however, females with weak fidelity outperformed females with strong fidelity during anomalous climate conditions (Abrahms et al. 2018). This suggests that maintaining multiple strategies within a population, such as individual generalists and specialists, can have population‐level benefits (Winemiller 1989; Codron et al. 2012; Abrahms et al. 2018; McHuron et al. 2018). Specifically, intraspecific variability found in leopard seals at Cape Shirreff may provide resilience to the changing ecosystem of the WAP.

Long‐term specialization by an apex predator can lead to sustained impacts on prey populations. At Cape Shirreff, leopard seals specializing in AFS pups have been proposed as the primary driver of the catastrophic decline in the local AFS population (Krause et al. 2022). Our study supports this hypothesis. First, we find that large adult females at Cape Shirreff are primarily high trophic‐level specialists. Second, some of these females show multi‐year patterns of specialization in nitrogen‐rich prey (i.e., AFS). Our findings match those described for orcas. Williams et al. (2004) estimated that a pod of five orcas specializing in sea otters ( *Enhydra lutris* ) could kill 8500 otters annually. Their model showed that if only ~4% of the 170 orca individuals around the Aleutian archipelago specialized in sea otters, they could drive the sea otters to extinction within 3–4 months (Williams et al. 2004). Likewise, at Cape Shirreff, a mean of only ~20 individual leopard seals have been responsible for the population collapse of AFS since 2007, with an estimated 69.3% (range: 50.3%–80.9%) of pups born being consumed by leopard seals (Krause et al. 2022). However, while leopard seal predation contributes to AFS population declines, competition with krill fisheries may also play a role by imposing both top–down and bottom–up pressures (Krause et al. 2024).

Looking across our study period, we also note that leopard seals at Cape Shirreff experienced a significant population‐level decline in δ^15^N values between 2015 and 2017. This decline in δ^15^N coincides with a decrease in the AFS population (Krause et al. 2022) and the two penguin species, gentoo ( *Pygoscelis papua* ) and chinstrap penguins ( *P. antarcticus* ), found breeding at this location (Hinke et al. 2019). Therefore, this decrease in δ^15^N may have been caused by a decline in nitrogen‐rich prey. Between 2017 and 2023, sightings of leopard seals at Cape Shirreff decreased by 76% (Krause et al. 2024; Woodman et al. 2024). This substantial drop in leopard seal numbers may explain the more recent increase in δ^15^N values (2018–2023), as reduced competition has allowed the few remaining leopard seals (e.g., seal 397) to continue specializing in the remaining high trophic‐level prey. Although previous studies have analyzed the isotopic values of available prey at Cape Shirreff (Krause et al. 2020), shifts in δ^15^N could also be influenced by changes in other ecological factors and variations at the base of the food web (Queirós et al. 2024). Nevertheless, together, these findings highlight the complexity of predator–prey dynamics and emphasize the importance of concurrently monitoring predator and prey populations to understand ecosystem‐level processes.

### 4.4 Conclusions and Considerations

Apex predators play a crucial role in shaping ecosystem dynamics, often exerting substantial influence on prey populations and resource distribution through their trophic interactions. The leopard seal population at Cape Shirreff demonstrates this influence, as it is composed mostly of individual specialists, with a few generalists. Long‐term patterns of high trophic‐level specialization have likely led to the decline of the AFS population at Cape Shirreff. Our study provides a compelling explanation for the dramatic impact a few specialist apex predator individuals can have on prey populations.

While our study utilized stable isotope analysis to assess trophic specialization, we did not directly measure diet through scat analysis, stomach contents, or direct foraging observations. As a result, our findings provide insights into trophic‐level specialization but do not identify specific prey species consumed by individual seals. We did not extensively analyze δ^13^C values due to the lack of tracking data, limiting our ability to interpret whether variability reflects habitat shifts, foraging specialization, or environmental changes. Future studies integrating stable isotope analysis with tracking data would provide a more comprehensive understanding of spatial foraging patterns in leopard seals. Additionally, while we tracked specialization across years, repeat samples were only available for a subset of individuals, meaning we cannot determine whether other seals maintained consistent foraging strategies over time. However, leopard seal samples are notoriously difficult to obtain, and this study represents the largest dataset to date, with the most repeat samples from individuals, including some sampled over four different years.

Although we focused on a single location, genetic studies on this aggregation indicate that it may be representative of the species (Bender et al. 2023), for which most populations appear to be genetically connected (Davis et al. 2008). Leopard seals are distributed across the Southern Ocean, including South America and New Zealand (Hupman et al. 2020; Borras‐Chavez et al. 2024), requiring future research to examine the degree of specialization across the species' range and how this impacts local prey populations. Our findings have far‐reaching implications, as many current ecosystem management and conservation strategies assume that most apex predators are generalists and that individuals exert similar effects on prey populations. However, if specialization patterns consistently vary among individuals, uniform conservation and management strategies could lead to unintended consequences in many ecosystems—a concern that has been previously recognized in pinniped populations (Villegas‐Amtmann et al. 2013; de Lima et al. 2019; Riverón et al. 2021). Our findings reinforce the need to reassess such strategies, particularly given the uncertain prey abundance for these predators in the face of changing climate conditions.

## Author Contributions

**Emily S. Sperou:** conceptualization (lead), data curation (lead), formal analysis (lead), funding acquisition (supporting), investigation (lead), methodology (lead), project administration (lead), resources (lead), software (lead), supervision (lead), validation (lead), visualization (lead), writing – original draft (lead), writing – review and editing (lead). **Douglas J. Krause:** conceptualization (supporting), data curation (lead), investigation (supporting), methodology (supporting), project administration (equal), writing – review and editing (equal). **Renato Borras‐Chavez:** conceptualization (supporting), data curation (equal), investigation (supporting), methodology (equal), project administration (supporting), resources (equal), writing – review and editing (equal). **Patrick Charapata:** conceptualization (supporting), investigation (supporting), methodology (equal), project administration (supporting), writing – review and editing (equal). **Daniel P. Costa:** data curation (equal), funding acquisition (supporting), project administration (supporting), writing – review and editing (equal). **Daniel E. Crocker:** data curation (equal), funding acquisition (supporting), project administration (supporting), writing – review and editing (supporting). **Kerri J. Smith:** investigation (supporting), methodology (equal), project administration (supporting), writing – review and editing (equal). **Bradley Thompson:** formal analysis (equal), investigation (equal), methodology (equal), writing – review and editing (supporting). **Azana Best:** data curation (equal), formal analysis (equal), methodology (supporting), writing – review and editing (supporting). **Jaelyn Anderson:** data curation (equal), formal analysis (equal), methodology (supporting), writing – review and editing (supporting). **Michael E. Goebel:** conceptualization (supporting), data curation (equal), investigation (equal), project administration (equal), resources (equal), writing – review and editing (equal). **Carolina A. Bonin:** conceptualization (equal), funding acquisition (lead), project administration (equal), writing – review and editing (equal). **Sarah S. Kienle:** conceptualization (equal), data curation (equal), funding acquisition (lead), investigation (equal), project administration (equal), writing – review and editing (equal).

## Conflicts of Interest

The authors declare no conflicts of interest.

## Supporting information


**Figure S1.** Clustering analysis showing two distinct groups of δ^15^N specialists.

**Figure S2.** Isotopic variation with sex and mass.

**Figure S3.** Isotopic signatures for each individual with multiple years of data (*n* = 7).

**Table S1.** Biologically relevant linear mixed‐effects candidate models used to explain variability in ẟ^15^N signatures of leopard seals whiskers using AICc for model selection.
**Table S2.** Table of coefficients, standard errors, and significance for our top linear models explaining the variance in mean ẟ^15^N (see Table S1).
**Table S3.** Quadratic discriminant analysis (QDA) results for individuals (*n* = 7) with whiskers sampled over multiple years.
**Table S4.** Percentage of niche overlap (SIBER), SEAc, and TA values for each year from individuals with repeat whisker samples (*n* = 7).
**Table S4.** Table of coefficients, standard errors (SE), confidence intervals (95% CI), *t*‐values, and *p*‐values for the intercept and smooth terms (Months, Year, Individual_ID) in the GAM model.

## Data Availability

The data supporting the findings of this study are available in the Dryad Digital Repository at https://doi.org/10.5061/dryad.f4qrfj75k.
