# Supplementary figures and images for: Individual Specialization in a Generalist Apex Predator: The Leopard Seal
Source: Ecol Evol. 2025 Jun 23;15(6):e71593. doi: 10.1002/ece3.71593 (PMC12184730; doi:10.1002/ece3.71593)

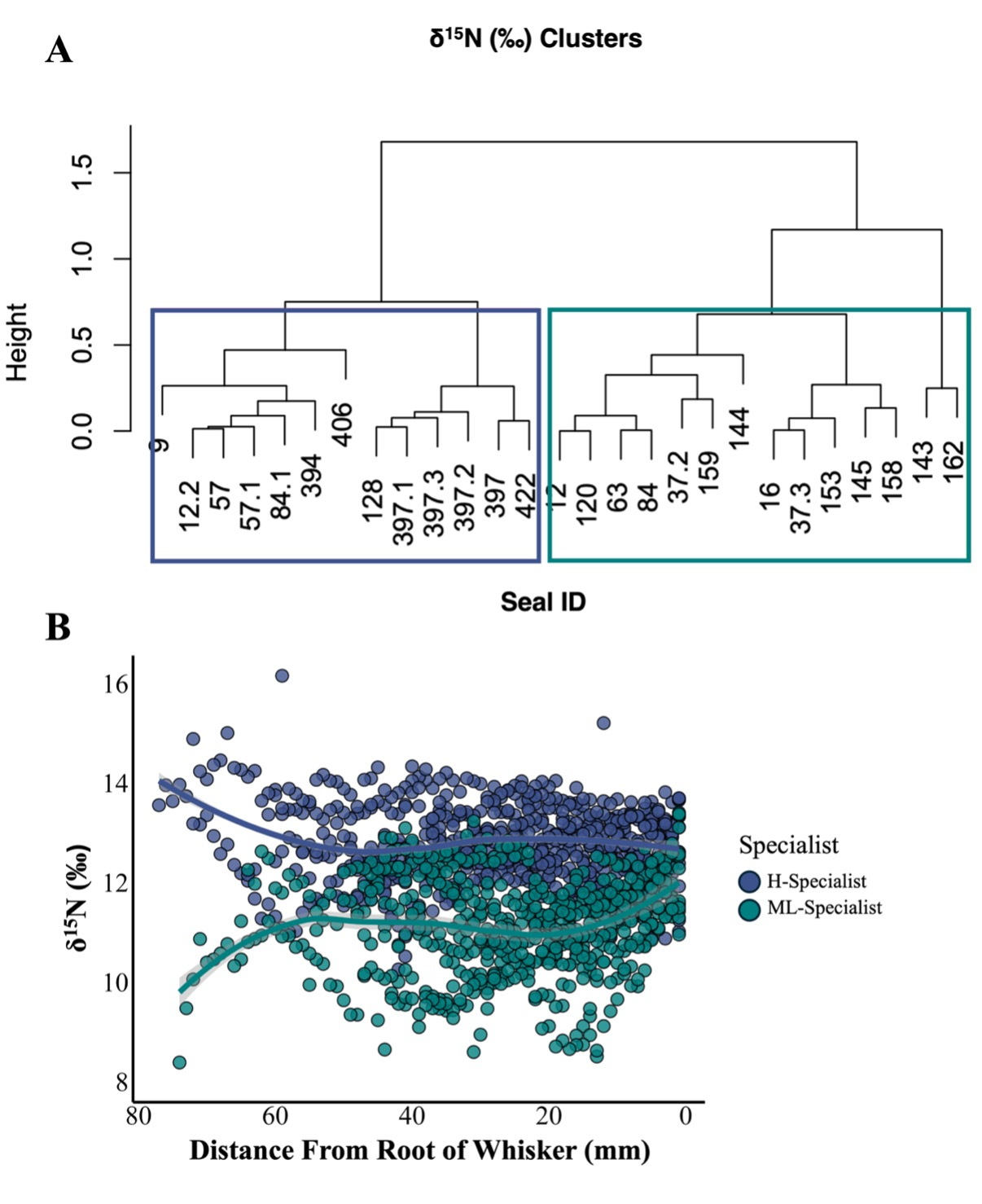

Supplement: Supplementary file 1 — Figure S1. Clustering analysis showing two distinct groups of δ15N specialists. [file ECE3-15-e71593-s002.tiff]

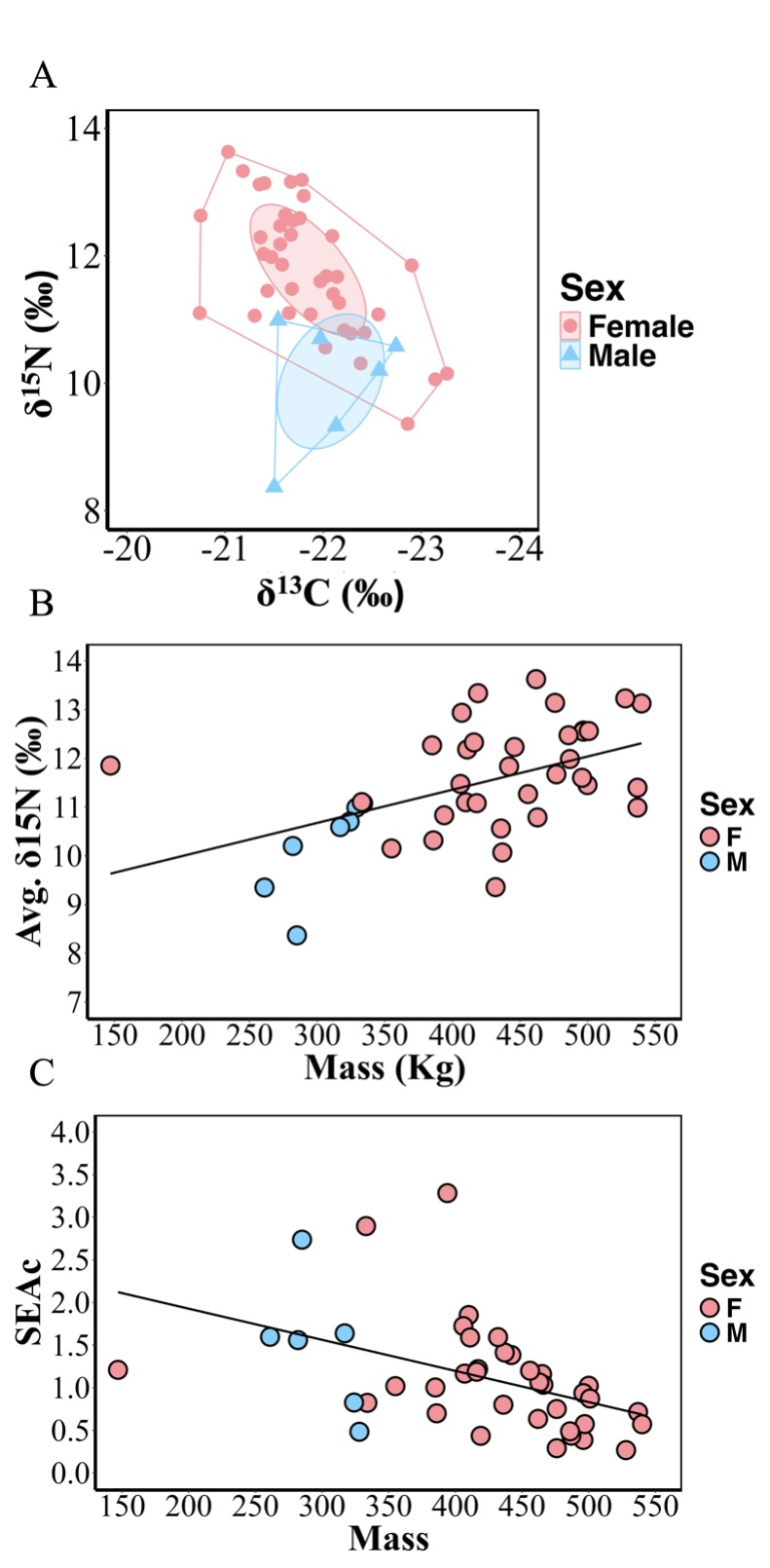

Supplement: Supplementary file 2 — Figure S2. Isotopic variation with sex and mass. [file ECE3-15-e71593-s001.tiff]

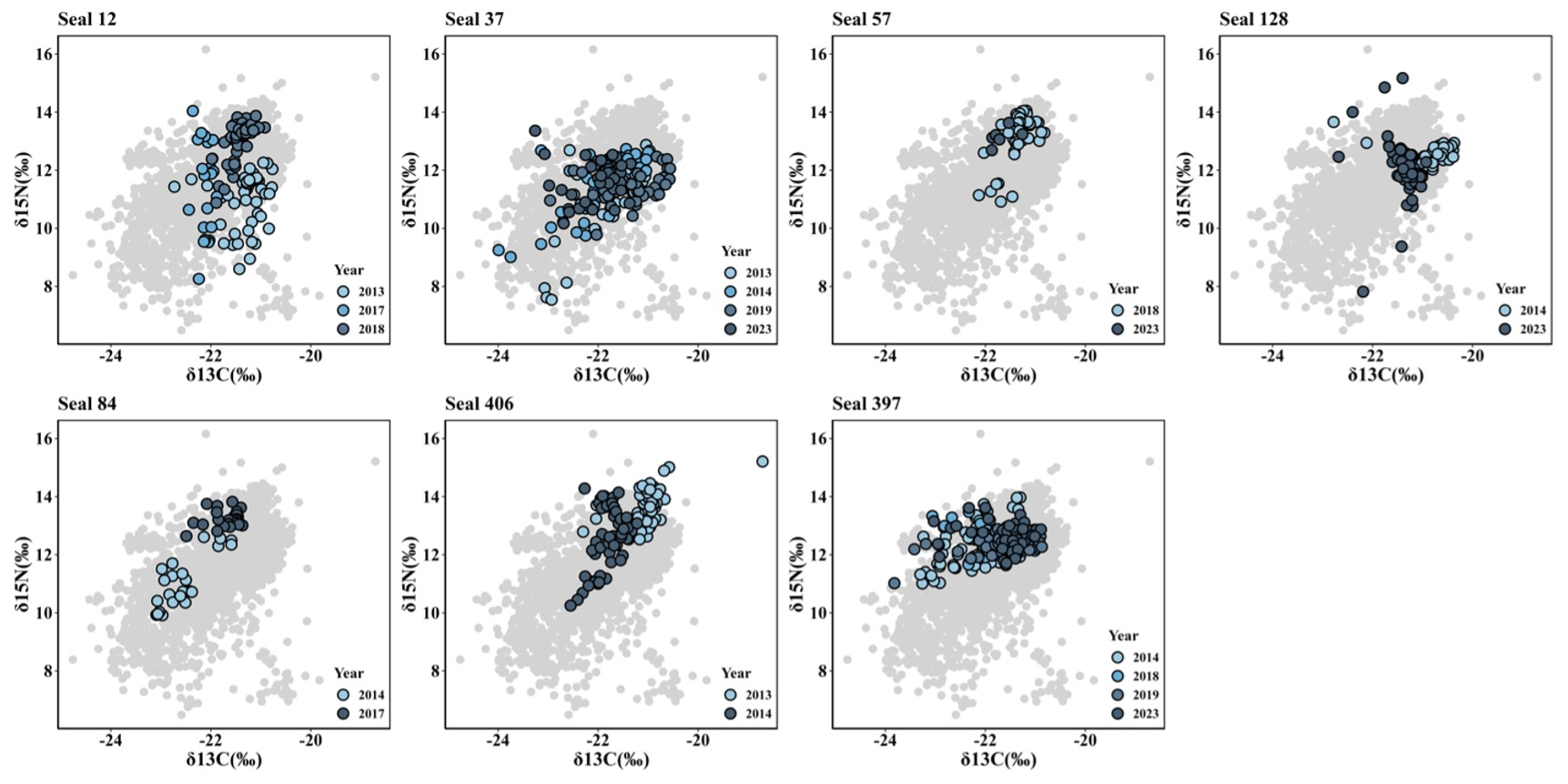

Supplement: Supplementary file 3 — Figure S3. Isotopic signatures for each individual with multiple years of data (n = 7). [file ECE3-15-e71593-s004.tiff]
