# Supplementary material for: Individual Specialization in a Generalist Apex Predator: The Leopard Seal
Source: Ecol Evol. 2025 Jun 23;15(6):e71593. doi: 10.1002/ece3.71593 (PMC12184730; doi:10.1002/ece3.71593)
Supplement: Supplementary file 4 — Table S1. Biologically relevant linear mixed‐effects candidate models used to explain variability in ẟ15N signatures of leopard seals whiskers using AICc for model selection. Table S2. Table of coefficients, standard errors, and significance for our top linear models explaining the variance in mean ẟ15N (see Table S1). Table S3. Quadratic discriminant analysis (QDA) results for individuals (n = 7) with whiskers sampled over multiple years. Table S4. Percentage of niche overlap (SIBER), SEAc, and TA values for each year from individuals with repeat whisker samples (n = 7). Table S4. Table of coefficients, standard errors (SE), confidence intervals (95% CI), t‐values, and p‐values for the intercept and smooth terms (Months, Year, Individual_ID) in the GAM model. [file ECE3-15-e71593-s003.docx]

**Individual Specialization in a Generalist Apex Predator: The Leopard Seal**

**Supplementary Material**

**Table S1**. Biologically relevant linear mixed effects candidate models used to explain variability in ẟ^15^N signatures of leopard seals whiskers using AICc for model selection. All models were created based on the full model. Here, K is the number of parameters, AICc is the Akaike information criteria for small sample sizes, ΔAICc represents the change in AICc from top selected model, AICc Wt is the proportion of predictive power compared to candidate model set, Cum. Wt is the cumulative predictive power, LL is the log-likelihood, R^2(c)^ is the proportion of variance explained by the fixed effects only, R^2(m)^ is the variance explained by both the random and fixed effect. Our top models (<2 AICc) are in bold.

| Model | K | AICc | ΔAICc | AICc Wt | Cum. Wt | LL | R2 (c) | R2(m) |
| --- | --- | --- | --- | --- | --- | --- | --- | --- |
| Null Model. ẟ^15^N ~ 1 + (1 \| Individual.ID) | 3 | 145.68 | 35.54 | 0.00 | 1.00 | -69.56 | 0 | 0.65 |
| 1. ẟ^15^N ~ Sex + (1 \| Individual.ID) | 4 | 137.09 | 26.94 | 0.00 | 1.00 | -64.06 | 0.22 | 0.63 |
| 1. ẟ^15^N ~ Mass + (1 \| Individual.ID) | 4 | 137.55 | 27.40 | 0.00 | 1.00 | -64.22 | 0.23 | 0.51 |
| 1. ẟ^15^N ~ ẟ^15^N Specialization + (1 \| Individual.ID) | 6 | 117.55 | 7.41 | 0.02 | 0.99 | -51.70 | 0.41 | 0.91 |
| 1. ẟ^15^N ~ Sex + Mass + (1 \| Individual.ID | 5 | 135.89 | 25.75 | 0.00 | 1.00 | -62.09 | 0.31 | 0.52 |
| 1. ẟ^15^N ~ Sex + ẟ^15^N Specialization + (1 \| Individual.ID) | **7** | **110.14** | **0.00** | **0.63** | **0.63** | **-46.60** | **0.58** | **0.92** |
| 1. ẟ^15^N ~ Mass + ẟ^15^N Specialization + (1 \| Individual.ID) | 7 | 114.02 | 3.88 | 0.09 | 0.97 | -48.31 | 0.51 | 0.97 |
| 1. ẟ^15^N ~ Sex + Mass + ẟ^15^N Specialization + (1 \| Individual.ID) | **8** | **112.01** | **1.87** | **0.25** | **0.88** | **-45.76** | **0.61** | **0.98** |
| 1. ẟ^15^N ~ Sex + Mass + Sex*Mass + (1 \| Individual.ID) | 6 | 143.06 | 32.91 | 0.00 | 1.00 | -64.29 | 0.34 | 0.53 |
| Full Model. ẟ^15^N ~ Sex + Mass + Sex*Mass + ẟ^15^N Specialization + (1 \| Individual.ID) | 9 | 118.08 | 7.93 | 0.01 | 1.00 | -47.14 | 0.64 | 0.98 |

**Table S2.** Table of coefficients, standard errors, and significance for our top linear models explaining the variance in mean ẟ^15^N (see Table S1).

| **Top Model: ẟ^15^N ~ Sex + ẟ^15^N Specialization + (1 \| Individual.ID)** | | | | |
| --- | --- | --- | --- | --- |
| **Coefficients** | Estimate | Std Error | t-value | p-value |
| **Intercept** | 10.98 | 0.24 | 44.47 | p < 0.001* |
| **SexM** | -1.21 | 0.35 | -3.44 | p = 0.001* |
| **H-Specialist** | 1.81 | 0.27 | 6.60 | p < 0.001* |
| **Intermediate** | 0.37 | 0.27 | 1.36 | p =0.18 |
| **ML-Specialist** | 0.21 | 0.25 | 0.86 | p = 0.4 |
| **Random Effects** | **Variance** | | **Std. Dev** | |
| **Individual.ID** | 0.49 | | 0.69 | |
| **Residual** | 0.10 | | 0.32 | |
| **Relevant Model: ẟ^15^N ~ Sex + Mass + ẟ^15^N Specialization + (1 \| Individual.ID)** | | | | |
| **Coefficients** | **Estimate** | **Std Error** | **t-value** | **p-value** |
| **Intercept** | 9.96 | 0.41 | 24.26 | p < 0.001* |
| **SexM** | -0.87 | 0.37 | -2.34 | 0.02* |
| **Mass** | 0.01 | 0.01 | 2.97 | 0.03* |
| **H-Specialist** | 1.78 | 0.16 | 10.77 | p < 0.001* |
| **Intermediate** | 0.35 | 0.17 | 2.05 | 0.09 |
| **ML-Specialist** | 0.50 | 0.15 | 0.33 | 0.75 |
| **Random Effects** | **Variance** | | **Std. Dev** | |
| **Individual.ID** | 0.59 | | 0.77 | |
| **Residual** | 0.02 | | 0.16 | |

**Table S3.** Quadratic Discriminant Analysis (QDA) results for individuals (n =7) with whiskers sampled over multiple years. The column 'n' refers to the total number of whisker segments analyzed for isotope data, and 'correctly assigned' indicates the number of isotope data points correctly attributed to their respective year.

| **Individual** | **Year** | **n** | **Average ẟ^13^C** | **Average ẟ^15^N** | **Correctly Assigned** | **% Correctly Assigned** |
| --- | --- | --- | --- | --- | --- | --- |
| *12* | 2013 | 47 | -21.29 | 10.98 | 45 | 96% |
|  | 2017 | 22 | -22.10 | 11.39 | 22 | 100% |
|  | 2018 | 61 | -21.41 | 13.14 | 56 | 92% |
| **Total Average Percent Classified** | **95%** | | | | | |
| *37* | 2013 | 36 | -21.65 | 11.09 | 12 | 33% |
|  | 2014 | 44 | -21.95 | 11.47 | 14 | 32% |
|  | 2019 | 47 | -21.43 | 11.45 | 31 | 66% |
|  | 2023 | 35 | -22.03 | 11.68 | 19 | 54% |
| **Total Average Percent Classified** | **46%** | | | | | |
| *57* | 2018 | 45 | -21.35 | 13.13 | 34 | 75% |
|  | 2023 | 6 | -21.67 | 13.16 | 5 | 83% |
| **Total Average Percent Classified** | **79%** | | | | | |
| *84* | 2014 | 23 | -22.56 | 11.07 | 22 | 96% |
|  | 2017 | 26 | -21.69 | 13.23 | 26 | 100% |
| **Total Average Percent Classified** | **98%** | | | | | |
| *128* | 2014 | 44 | -20.81 | 12.57 | 42 | 95% |
|  | 2023 | 47 | -21.38 | 12.03 | 40 | 85% |
| **Total Average Percent Classified** | **90%** | | | | | |
| *397* | 2014 | 77 | -22.11 | 12.27 | 37 | 48% |
|  | 2018 | 46 | -21.69 | 12.55 | 22 | 47% |
|  | 2019 | 62 | -21.57 | 12.47 | 38 | 61% |
|  | 2023 | 49 | -21.61 | 12.63 | 14 | 28% |
| **Total Average Percent Classified** | **46%** | | | | | |
| *406* | 2013 | 71 | -21.02 | 13.63 | 67 | 94% |
|  | 2014 | 56 | -21.80 | 12.56 | 51 | 91% |
| **Total Average Percent Classified** | **93%** | | | | | |

**Table S4.** Percentage of niche overlap (SIBER), SEAc, and TA values for each year from individuals with repeat whisker samples (n =7).

| **Individual ID** | **Year** | **SEAc** | **TA** | **Overlap (SEAc) Between Years** |
| --- | --- | --- | --- | --- |
| *12* | 2013 | 1.16 | 4.15 | 2013/2017: 0% |
|  |  |  |  | 2017/2013: 0% |
|  | 2017 | 0.71 | 2.05 | 2013/2018:0% |
|  |  |  |  | 2018/2013: 0% |
|  | 2018 | 0.29 | 1.42 | 2017/2018: 0% |
|  |  |  |  | 2018/2017: 0% |
| **Average Overlap Between Years** | **0%** | | | |
| *37* | 2013 | 1.85 | 5.71 | 2013/2014: 37% |
|  |  |  |  | 2014/2013: 40% |
|  |  |  |  | 2013/2019: 43% |
|  | 2014 | 1.72 | 7.01 | 2019/2013:78% |
|  |  |  |  | 2014/2019: 28% |
|  |  |  |  | 2019/2014: 47% |
|  | 2019 | 1.02 | 4.02 | 2013/2023: 17% |
|  |  |  |  | 2023/2013: 26% |
|  |  |  |  | 2014/2023: 54% |
|  | 2023 | 1.18 | 3.85 | 2023/2014: 78% |
|  |  |  |  | 2019/2023: 30% |
|  |  |  |  | 2023/2019: 26% |
| **Average Overlap Between Years** | **42%** | | | |
| *57* | 2018 | 0.57 | 2.45 | 2018/2023: 9% |
|  | 2023 | 0.23 | 0.26 | 2023/2018: 22% |
| **Average Overlap Between Years** | **16%** | | | |
| *84* | 2014 | 0.82 | 1.93 | 2014/2017: 0% |
|  | 2017 | 0.26 | 0.84 | 2017/2018: 0% |
| **Average Overlap Between Years** | **0%** | | | |
| *128* | 2014 | 0.38 | 1.53 | 2014/2023: 10% |
|  | 2023 | 1.21 | 7.96 | 2023/2014: 3% |
| **Average Overlap Between Years** | **7%** | | | |
| *397* | 2014 | 1.00 | 3.99 | 2014/2018: 33% |
|  |  |  |  | 2018/2014: 58% |
|  |  |  |  | 2014/2019: 23% |
|  | 2018 | 0.57 | 2.50 | 2019/2014: 47% |
|  |  |  |  | 2014/2023: 31% |
|  |  |  |  | 2023/2014: 42% |
|  | 2019 | 0.49 | 2.63 | 2018/2019: 56% |
|  |  |  |  | 2019/2018: 65% |
|  |  |  |  | 2018/2023: 91% |
|  | 2023 | 0.73 | 3.09 | 2023/2018: 71% |
|  |  |  |  | 2019/2023: 81% |
|  |  |  |  | 2023/2019: 54% |
| **Average Overlap Between Years** | **54%** | | | |
| 406 | 2013 | 0.63 | 0.87 | 2013/2014: 0% |
|  | 2014 | 4.51 | 3.08 | 2014/2013: 0% |
| **Average Overlap Between Years** | **0%** | | | |

**Table S4.** Table of coefficients, standard errors (SE), confidence intervals (95% CI), t-values, and p-values for the intercept and smooth terms (Months, Year, Individual_ID) in the GAM model. The smooth terms show the F-values, degrees of freedom (df), and significance levels, indicating the contribution of each term to the model variance.

| **Parameter** | **Coefficient** | **SE** | **95% CI** | **t(2145.09)** | **p** |
| --- | --- | --- | --- | --- | --- |
| Intercept | 11.49 | 0.17 | [11.15, 11.82] | 67.68 | < .001 |
| **Smooth Terms** | | | **F** | **df** | **p** |
| Months | | | 35.35 | 3.60 | 0.079 |
| Year | | | 7.03 | 6.96 | < 0.001 |
| Individual_ID | | | 51.43 | 42.35 | < 0.001 |

**Figure S1.** Clustering analysis showing two distinct groups of δ¹⁵N specialists. (**A**) Hierarchical clustering dendrogram showing two clusters of seals based on δ¹⁵N values. The clusters are determined using the Dunn index, which identifies the two optimal clusters for the data set: high trophic level specialists (H-Specialist; n = 13); medium to low trophic level specialist (ML-Specialist; n = 14). (**B**) Scatter plot of δ¹⁵N isotopic data along the whisker length for each individual seal. Each point represents a δ¹⁵N measurement at a specific distance from the root of the whisker. Seals are color-coded according to their δ¹⁵N specialization category: H-Specialist (blue) and ML-Specialist (green). The smooth trends represent the average δ¹⁵N values for each specialization category, highlighting the differences in isotopic patterns between the two groups.

**Figure S2**. Isotopic variation with sex and mass. (**A**) Bivariate plot of isotopic space (δ^15^N vs. δ^13^C) for male (blue triangles) and female (pink circles) leopard seals. The ellipses represent the standard isotopic niche area for each sex, with polygons outlining the convex hull encompassing all individual data points. (**B**) Linear regression of the relationship between average δ^15^N and body mass (kg) for male (blue) and female (pink) leopard seals (y = 8.23 – 0.007x, R^2^ = 0.28, p = 0.001). (**C**) Linear regression of the relationship between standard ellipse area corrected for small sample sizes (SEAc) and body mass (kg) for male (blue) and female (pink) leopard seals y=2.66 − 0.004x, R^2^ =0.21, p = 0.02).

**Figure S3.** Isotopic signatures for each individual with multiple years of data (n=7). Light grey dots represent population-level isotopic signatures, while variations in blue indicate different years.
